# Nutrition Behavior and Physical Activity of Middle-Aged and Older Adults in Saudi Arabia

**DOI:** 10.3390/nu14193994

**Published:** 2022-09-26

**Authors:** Rouba Khalil Naaman

**Affiliations:** Clinical Nutrition Department, Faculty of Applied Medical Sciences, King Abdulaziz University, P.O. Box 80215, Jeddah 21589, Saudi Arabia; rnaaman@kau.edu.sa

**Keywords:** older adults, middle-aged, nutrition behavior, dietary pattern, physical activity

## Abstract

As people get older, their nutritional status deteriorates, resulting in increased vulnerability to chronic diseases. The adoption of a healthy lifestyle has been linked to improved health throughout the aging process. The current study aimed to assess nutritional behaviors, dietary patterns, and physical activity among middle-aged and older adults in Saudi Arabia. An electronic questionnaire was completed between September and November 2021 by 419 participants aged 45 years and older. Of those, 65% reported that nutrition was important to them and 19% stated that they were consuming a healthy diet. Participants reported consuming an average of around 6 servings/week each of fruit and vegetables, with mean intake scores of 5.92 ± 0.25 and 5.57 ± 0.22, respectively. It was reported that around 3 servings/week of red meat, 4 servings/week of poultry, and 1 serving/week of fish were consumed, with mean intake scores of 2.65 ± 0.13, 4.34 ± 0.16, and 1.36 ± 0.08, respectively. Most of the participants (60%) reported being inactive. Middle-aged and older adults living in Saudi Arabia have poor dietary patterns and nutritional behaviors. Education and guidance on nutrition are needed for this population to help them improve their diet and lifestyle.

## 1. Introduction

The percentage of the world’s population that is elderly is growing rapidly, and it is expected that there will be 1.4 billion adults aged 60 years and over by 2050 [1]. According to the United Nations 2021, in Saudi Arabia, the percentage of the country’s population that is elderly increased from 4.5% in 2010 to 5.9% in 2020, and this is estimated to further increase in the coming years [2]. This ageing of the population comes with challenges that need to be considered by governments and health services, including an increased risk of chronic illnesses and co-morbidities, and consequent increased healthcare costs.

As people get older, their nutritional status deteriorates as a result of several physiological and social age-related changes. Deteriorated digestive function, loss of muscle mass, impaired functional status, loss of appetite, poor oral health, depression, social isolation, and poor economic status are some examples of changes that often accompany aging, which, in turn, result in poor health outcomes such as increased vulnerability to chronic diseases, infections, physical disability, and cognitive impairments [1,3,4]. Healthy aging refers to the maintenance of physical and cognitive health with a reduced risk of chronic diseases with aging [5]. The link between a healthy lifestyle and healthy aging has recently been extensively studied [6,7]. The adoption of healthy habits such as the consumption of a balanced diet and engagement in physical activity has been shown to be associated with improved overall health and functional capacity in old age [8].

The quality of one’s diet is one of the key factors that can be modified to prevent the development of chronic diseases with aging. A previous study that assessed the intake of Saudi adults aged 15 and older showed that only a small percentage of the Saudi population met the Saudi dietary guidelines [9]. However, there is still a paucity of studies that assess the dietary behavior of middle-aged and older adults’ in Saudi Arabia. Therefore, to help fill this gap in the research, the current study aims to assess nutritional behaviors, dietary patterns, and physical activity in middle-aged and older adults in Saudi Arabia. 

## 2. Materials and Methods

### 2.1. Study Design

This cross-sectional study was approved by the Unit of the Biomedical Ethics Research Committee at King Abdulaziz University (Jeddah, Saudi Arabia) (reference no. 426-21). A convenience sample of 419 participants was recruited electronically and asked to complete an online questionnaire. All study participants gave their consent to participate in this study at the beginning of the online questionnaire.

### 2.2. Participants and Recruitment

The inclusion criteria were being a male or female citizen or resident of Saudi Arabia aged ≥45 years or older. Between September and November 2021, the study participants completed an electronic questionnaire. The questionnaire was created via Microsoft Forms, version Microsoft 365 (Microsoft Corporation, Redmond, WA, USA) and distributed on WhatsApp (Facebook, Inc., Menlo Park, CA, USA) and Twitter (Twitter, Inc., San Francisco, CA, USA). WhatsApp was used to share the questionnaire link with the authors’ relatives and friends so that they could participate in the study and share the questionnaire link with their own contacts. The study’s information and participation link were also posted on Twitter, and to reach a wider group of people from various regions of Saudi Arabia, the link to the questionnaire was promoted. 

### 2.3. Questionnaire

This online questionnaire was intended to assess dietary and nutritional behaviors and physical activity in middle-aged and older adults in Saudi Arabia. To improve the quality of the questionnaire, three nutrition experts (MSc and PhD holders) reviewed the questionnaire in terms of relevance, clarity, simplicity, and ambiguity, and their comments on it were taken into account. Modifications and changes that were made included correcting linguistic and grammatical errors, rewording, and adding options to some questions regarding participants’ sociodemographic characteristics and dietary patterns. For pre-testing, the revised questionnaire was shared with four persons aged 45 and over (who were not included in the main study) so that they could check the understandability of the questions and answers, and they could assess the average time needed for completion. Changes were then made based on the pre-testing’s feedback, including revising and reformulating some questions and their options. As a result of these amendments, the final version of the questionnaire, which needed around 15 min to complete, consisted of four main sections. It was created and distributed in Arabic. Participants were provided with information regarding the study at the beginning of the questionnaire, including the study’s main aims and inclusion criteria, the confidentiality of the data being collected, and the expected time needed to complete the questionnaire.

In section one, participants’ socio-demographic data were collected, including age, gender, nationality, marital status, city of residence, work status, educational level, monthly income, living status, presence of chronic disease, and smoking status. Participants’ anthropometric measurements, including self-reported weight in kilograms and height in centimeters, were also collected. Both measurements were then used to determine their body mass index (BMI). 

In section two, participants were asked about how important nutrition was to them and to rate the healthfulness of their diet by selecting one of the following options: important/healthy, somewhat important/somewhat healthy, or not important/not healthy. Questions about whether they received any dietary advice or information related to dietary intake and the source of the advice were also included. Regarding nutrition facts labels, participants were asked about how frequently they read such labels when buying new food products and, if they indicated that they did not read them, why not.

In section three, participants were asked about their consumption of certain food groups, including starch, fruits, vegetables, milk and dairy products, red meat, poultry, fish, and legumes. For each food group, participants indicated the frequency of their consumption of the group by choosing one of the following options: I do not eat it at all, 1–3 times/month, 1 time/week, 2 times/week, 3 times/week, 4 times/week, 5 times/week, 6 times/week, 1 time/day, or 2 or more times/day. Second, they were asked about the estimated portion size they would eat each time they consumed a given food group and were given the following answers to choose from: I do not eat it at all, <1 portion, 1 portion, 2 portions, 3–4 portions, or 5 or more portions. For each food group, participants were provided with examples of food items and the estimated size of one portion (e.g., starch: ½ cup cereals or 1 slice bread or 1 potato; fruits: 1 fruit or ½ cup juice or dried fruits; vegetables: 1 cup fresh or ½ cup cooked; milk and dairy products: 1 glass or cup or 1 slice of cheese; red meat/poultry/fish: 30 g; and legumes: 1 cup). To enable a better understanding of the portion sizes, pictures of the size of one portion were given for each food group. 

In section four, participants were asked whether they performed physical activity. They were also asked about the frequency and type of physical activity they performed per week, and those who did not perform any type of physical activity were asked why not.

### 2.4. Data Analysis

The frequency of intake of each food group and the portion size consumed each time a given food group was consumed were all converted to the number of servings consumed per week. The frequency of the food groups’ intake data was coded as follows: I do not eat it at all = 0, 1–3 times/month = 0.5, 1 time/week = 1, 2 times/week = 2, 3 times/week = 3, 4 times/week = 4, 5 times/week = 5, 6 times/week = 6, 1 time/day = 7, or 2 or more times/day = 14. Data about the portion size consumed each time of consumption were coded as the following: I do not eat it at all = 0, <1 portion = 0.5, 1 portion = 1, 2 portions = 2, 3–4 portions = 3, or 5 or more portions = 5. To obtain the number of servings consumed each week, the scores of frequency were multiplied by the scores of the consumed portion sizes. Then, the consumption of each food group was rated on a 7-point scale. A score of 0 was given when no consumption was reported, a score of 0.5 when <1 serving/week was reported, a score of 1 when 1 serving/week was reported, 2 for 2–3 servings/week, 4 for 4–6 servings/week, 7 for 7 servings/weeks or 1 serving/day, and a score of 14 when ≥14 servings/week or more or ≥2 servings/day were reported. 

### 2.5. Sample Size Calculation

The study sample size was computed by using Raosoft^®^ software, version 2004 (www.raosoft.com/samplesize.html) (Raosoft, Inc., Seattle, WA, USA) (accessed on 12 November 2021). The required sample size was calculated based on the population living in Saudi Arabia (aged ≥45 years old) as reported by the Saudi General Authority for Statistics in 2020 [10]. The anticipated frequency is 50%, with a 95% confidence level, a 5% margin error, and a design effect of 1; therefore, a minimum sample of 385 participants was required to be enrolled. 

### 2.6. Statistical Analysis

A statistical analysis was performed using Minitab^®^ statistical software, version 19 (Penn State University, State College, PA, USA). The Anderson–Darling test was used to evaluate the distribution of the variables. Categorical data were expressed as a number and a percentage, and continuous data were expressed as mean and standard deviation. Differences between categorical variables were assessed with the Chi-square test. The Kruskal–Wallis test was used to test the differences between continuous variables. A *p* value of <0.05 was considered to be statistically significant. 

## 3. Results

### 3.1. Characteristics of the Study Participants

This study’s questionnaire was completed by 419 participants. Table 1 presents the general characteristics of the population studied. Forty-six percent of the participants were aged 45–54 years old, 66% were males, 91% were Saudis, and 63% of them were from the Western Region of Saudi Arabia. The majority of the participants were married (86%), lived with others (62%), and received university-level education (61%). Forty-four percent of the recruited participants were retired, while 38% of them were employed. Most of them had monthly incomes higher than 10,000 Saudi riyals (66%).

Regarding the participants’ main medical diagnoses, 37% reported that they were not suffering from any medical disorder, and 34% reported that they had heart diseases. Most of the participants were nonsmokers (61%). Forty-one percent of them reported being overweight and thirty-eight percent being obese.

### 3.2. Dietary Behavior Self-Evaluation

The majority of the study participants (65%) reported that nutrition was important to them. A significant difference was seen with monthly income (*p* < 0.05). However, no differences were found with other variables. Regarding participants’ self-evaluation of the healthfulness of their diet, most of them (66%) stated that their diet was somewhat healthy and 19% stated that they were consuming a healthy diet. Significant differences were seen with age and monthly income (*p* < 0.05), whereas other variables such as gender, marital status, living situation, education, and work status showed no associations. The participants’ nutrition behavior self-assessment is presented in Table 2. 

**Figure 1:**
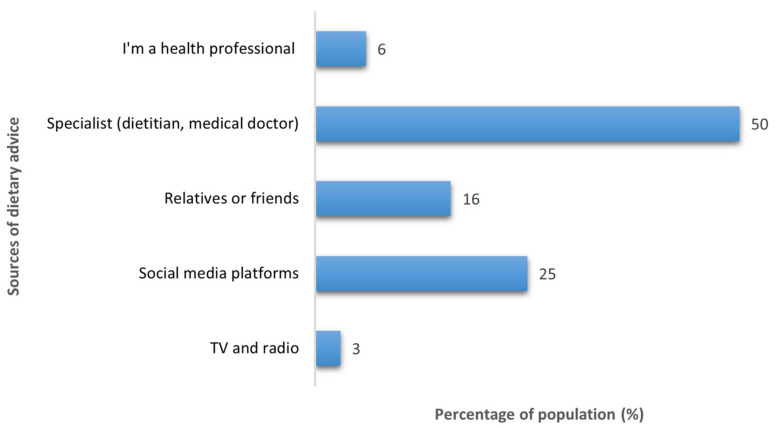
Participants’ sources of dietary advice (*n* = 252).

Sixty percent of the study participants reported that they had received dietary advice or information related to their dietary intake previously, with half of them (50%) stating that the source of this advice was from a health specialist, including dietitians and physicians. A quarter (25%) of those who reported that they had previously received dietary advice indicated that the source of the advice was from social media platforms such as Instagram, Twitter, Snapchat, and Facebook (Figure 1). 

**Figure 2:**
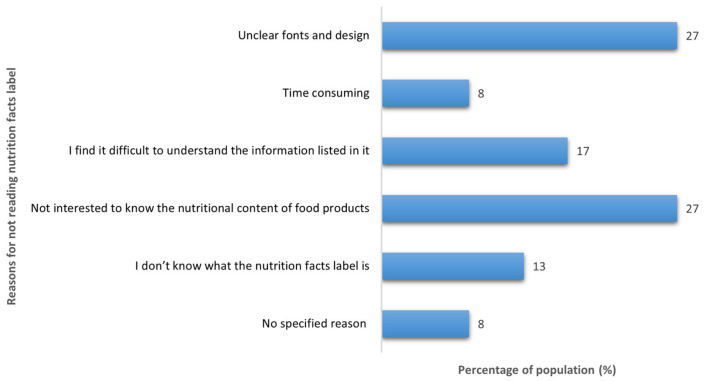
Reasons for not reading nutrition facts labels (*n* = 419).

Regarding reading nutrition facts labels when buying new food products, around half of the participants (51%) stated that they did not read the labels. Unclear fonts and designs and a lack of interest in knowing the nutritional content of food products were two of the most common reasons reported for not reading the nutrition facts label (Figure 2). For those who stated that they read the labels (49%), 53% of them said that they read it most of the time, 29% that they sometimes read it, and only 18% that they always read the labels. 

### 3.3. Dietary Intake Assessment

Table 3 shows the scores of the weekly intake of different food groups based on the frequency and quantity consumed. Across all study participants, the mean score for starch intake per week was 8.09 ± 0.24. There were no variables associated with starch intake (*p* > 0.05). With regard to fruits, the total mean score of weekly intake was 5.92 ± 0.25. Age, living situation, work status, and income were significantly associated with fruit consumption (*p* < 0.05). The mean score of vegetable intake was 5.57 ± 0.22, with age and gender being the variables associated with vegetable intake (*p* < 0.05). The total mean score of weekly intake of milk and dairy product was 5.56 ± 0.23. There were no variables associated with intake of milk and dairy products (*p* > 0.05). In terms of intake of red meat and poultry, the mean weekly intake scores were 2.65 ± 0.13 and 4.34 ± 0.16, respectively. Gender, marital status, work status, and income were variables associated with red meat and poultry intakes (*p* < 0.05). The mean score for weekly fish intake was 1.36 ± 0.08. Age, gender, marital status, work status, and income were all significantly associated with fish intake (*p* < 0.01). With regard to legumes, the total mean score of the intake per week was 2.03 ± 0.12. Gender and income were the variables that were found to be significantly associated with legume intake (*p* < 0.05).

### 3.4. Physical Activity Assessment

**Figure 3:**
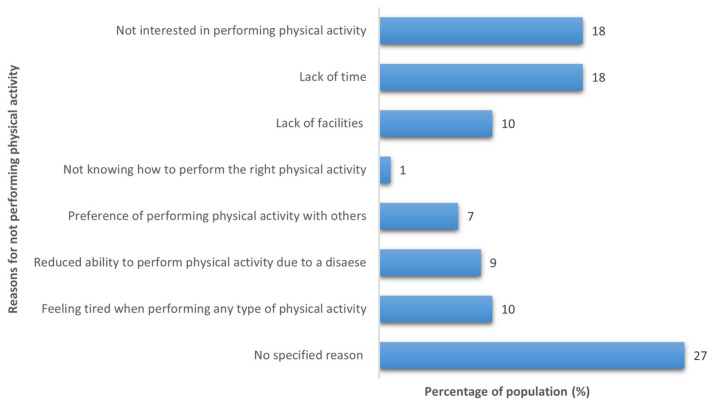
Reasons for not performing physical activity (*n* = 252).

Out of 419 participants, 167 (40%) reported that they performed physical activity, with 97 participants (58%) performing physical activity at least 5 times a week. Different types of physical activity were performed by the study participants, including walking (69%), swimming (9%), resistance exercise (6%), cardio exercise (4%), cycling (3%), running (3%), yoga (2%), and other types of physical activities (4%), such as football and dancing. For those who reported not performing physical activity (60%), the majority of them (27%) stated that they did not have any specific reason for not performing physical activity (Figure 3). 

## 4. Discussion

The association between diet and health status among older adults has been extensively assessed in previous studies. It has been shown that nutrition plays an important role in promoting health and preventing disease in this population [11]. However, in Saudi Arabia, the nutritional and dietary behaviors of middle-aged and older adults are not well identified. Hence, conducting this study is an important initial step to better understand the dietary patterns and behaviors of this population group, and will allow the influence of diet on the development of diseases to be better assessed in the future. Overall, the current study has shown that middle-aged and older adults living in Saudi Arabia are adopting poor dietary patterns and nutritional behaviors.

The majority of this study’s participants (95%) reported that nutrition and consuming a healthy diet were important or somewhat important to them, and a large percentage (85%) rated the healthfulness of their diet as healthy or somewhat healthy. Similarly, Gille et al., who assessed eating patterns and behavior in the Swiss middle-aged and elderly population, reported that 96% of the participants said that nutrition and healthy eating were important to them, with the majority overrating their diet as healthy [12]. 

Most participants in this study reported that they had previously received dietary advice and reported that dietitians and physicians were the first ones to be consulted in the event of any dietary concern. Dietitians are known for their experience in nutrition and dietetics and their extensive knowledge and training in this regard, and, as such, they were expected by the public to be the first source of dietary advice [13]. Consistent with previous studies, health professionals have been shown to be the most preferred source of dietary advice [14,15]. Recent studies have reported the increased use of social media platforms for health information [16,17]. Although people usually perceive the lack of accuracy in the information obtained from the internet and social media, speed, cheapness, and ease of access could be potential factors for those looking for nutrition information [18]. This is in line with the current study’s findings, which showed that a quarter of the participants accessed nutritional information by the use of social media platforms. These results suggest the importance of adopting new strategies to provide appropriate nutrition information by health professionals. For instance, virtual consultations can be used by dietitians as a more convenient and quick approach in place of face-to-face appointments.

Around half of the participants in the present study reported reading nutrition facts labels when buying new food items, with only 18% of those stating that they always read them. In contrast, a recent study was conducted among Saudi adults aged 18 years and over that showed that 62% of the studied population reported using nutrition facts labels when buying food products [19]. A possible explanation for the lower percentage of nutrition fact label usage in this study is due to the age of the recruited participants. In fact, 54% of this study participants were aged 55 years old and older, while the majority of the other study participants were younger, with only 7% of them aged 56 years or older. The influence of aging could be one of the factors that affect participants’ ability to understand or to interpret the information provided in the labels, which, in turn, will reduce the use of the information of food labels [20]. The present study supports this explanation and showed that unclear fonts and designs and difficulty in understanding the content of nutrition facts labels limited the use of the nutrition facts labels by a large number of the participants. This highlights the need to use other formats that might enhance consumers’ comprehension of the information provided on nutrition facts labels. Additionally, campaigns to increase awareness of how to use the information provided in the labels could be conducted, thereby guiding the consumer toward healthier and better food choices. 

One of the main aims of the current study was to assess the dietary patterns of the study population based on the frequency and quantity of consumption of different food groups. This study showed that the average fruit and vegetable consumption was six servings a week each, which is much lower than the intake recommended by the World Health Organization (WHO), which recommends at least five servings of fruits and vegetables to be consumed on a daily basis [21]. The low consumption of fruits and vegetables in this study population could be due to several barriers that might affect their consumption rates as they age. These factors include social isolation, poor dental health, and increased disease susceptibility [22]. Age was one of the factors affecting the consumption of fruits and vegetables in this study, with higher intakes at older ages. Similarly, it has been previously shown that individuals tend to increase their fruit and vegetable consumption as they age [22,23,24]. Fruits in Saudi Arabia are costly, and this could deter a large number of the population from purchasing fruits on a regular basis, particularly those with low monthly incomes [9,25]. This was reported in this study, where those with an income of higher than 10,000 riyals per month (around $2600), consumed significantly more servings of fruits compared with those with a lower income. An American study that assessed the effect of income on older adults’ eating patterns showed that those with low and medium incomes consumed significantly fewer fruits than those with higher incomes [26]. For that reason, it was previously estimated that increased income along with reduced fruit prices would likely lead to an increase in the rate of fruit consumption in the world’s elderly population [27].

Recent studies have highlighted the importance of protein adequacy for preserving muscle mass and function [28]. However, the adverse impact of meat overconsumption, red meat in particular, has been reported and it has been shown that moderation is the key for healthy aging [29]. Participants in this study exceeded the WHO-recommended weekly intake of red meat and poultry [21] while fish intake was below the WHO’s recommendations with an average weekly intake of three servings of red meat, four servings of poultry, and one serving of fish. Gender significantly influenced red meat, poultry, and fish consumption in the current study, with higher scores for men. This finding is in line with Saudi studies that have assessed dietary pattern in Saudi adults and shown that red meat and fish consumption was higher in men compared to women [9,30]. This could be explained by the fact that women often show greater dietary knowledge and therefore make better food choices than men [31,32]. Moreover, intakes of red meat, poultry, and fish were influenced by other factors in this study population, including monthly income. The average consumption was higher in those with higher monthly incomes, and this was similarly reported previously [33,34].

In Saudi Arabia, the prevalence of inactivity has always been a challenge to public health, as was shown in a study that assessed the performance of physical activity among adults aged 15 years old and over living in Saudi Arabia and showed that performance of physical activity was low and decreased with age, with around 88% of the population aged 45 and over reporting being inactive [35]. The present study supports this finding, showing a low level of physical activity, with 60% of the study participants stating that they did not perform physical activity.

Despite the importance of the present study in providing insight into nutritional behaviors and dietary patterns in middle-aged and older adults in Saudi Arabia, the study has some limitations. To allow for larger sample size recruitment and to obtain responses from various demographic populations, data in the current study were collected by distributing the questionnaire electronically. However, this approach could introduce a slight bias in the study results as it might not represent the actual sociodemographic structure of the entire population in Saudi Arabia. Another limitation is that participants in this study were asked to self-report the frequencies and quantities of different food groups’ intake rather than measuring the actual dietary intake, and this could increase the chance of intake misreporting.

## 5. Conclusions

Adopting healthy habits has been linked with improved health and wellbeing as people get older. Middle-aged and older adults living in Saudi Arabia seem to have poor dietary patterns and nutritional behaviors. While the majority of this study population considered nutrition to be important to them, a great percentage of them reported not using nutrition facts labels, having poor consumption levels of fruits, vegetables, fish, and legumes, and engaging in little physical activity. Therefore, implementing effective strategies to enhance knowledge of the importance of nutrition among the older population is needed. Nutrition education and guidance are warranted for middle-aged and older adults to help them improve their dietary choices and overall lifestyles as they get older. Likewise, there is a need for further studies assessing the actual dietary intake of different food group populations in Saudi Arabia with larger sample sizes, as well as comparisons with Saudi dietary recommendations.

## Figures and Tables

**Table 1:** General characteristics of the study participants (*n* = 419) ^1^.

Variables	N	%
Age (years)		
45–54	194	46
55–64	169	40
≥65	56	14
Mean ± SD	55.5 ± 7	
Gender		
Male	278	66
Female	141	34
Nationality		
Saudi	382	91
Non-Saudi ^2^	37	9
Region		
Western Region	262	63
Central Region	98	23
Eastern Region	33	8
Northern Region	9	2
Southern Region	17	4
Marital status		
Single	24	6
Married	362	86
Divorced	21	5
Widower	12	3
Living situation		
Living alone	158	38
Living with others	261	62
Education level		
High school education or less	81	19
University education	253	61
Higher education	85	20
Work status		
Employed	161	38
Freelance job	27	7
Retired	184	44
Unemployed	47	11
Income (SR)		
<2000	37	9
2000–5000	34	8
5000–7000	23	6
7000–10,000	48	11
>10,000	277	66
Main medical diagnosis		
No diseases	153	37
Heart diseases	144	34
Respiratory disorders	25	6
Gastrointestinal diseases	29	7
Cancer	10	3
Renal disorders	9	2
Liver disorders	4	1
Diabetes	102	24
Iron deficiency anemia	19	4
Osteoporosis	29	7
Others ^3^	23	5
Smoking		
Yes	93	22
No	257	61
Ex-smoker	69	17
BMI category ^4^		
Underweight	4	1
Normal	84	20
Overweight	171	41
Obese	160	38

^1^ Data presented as number and percentage. ^2^ Non-Saudis including Egyptian, Palestinian, Yemeni, Sudanese, and Jordanian. ^3^ Other diseases including hypothyroidism, gout, and multiple sclerosis. ^4^ Self-reported weight and height used to calculate the BMI. The BMI categories are underweight (<18.5 kg/m^2^), normal weight (18.5–24.9 kg/m^2^), overweight (25.0–29.9 kg/m^2^), and obese (≥30 kg/m^2^). Abbreviations: SD, standard deviation; SR, Saudi Riyals; BMI, body mass index.

**Table 2:** Nutrition behavior self-assessment (*n* = 419) ^1^.

Variables	How Important is Nutrition for You?			How Would You Rate the Healthfulness of Your Overall Diet?		
	Important	Somewhat Important	Not Important	Healthy	Somewhat Healthy	Not Healthy
Total (%)	65	30	5	19	66	15
Age (years)						
45–54	122 (29)	59 (14)	13 (3)	30 (7)	125 (30)	39 (9)
55–64	110 (26)	52 (12)	7 (2)	38 (9)	116 (28)	15 (4)
≥65	40 (10)	15 (4)	1 (0)	14 (3)	34 (8)	8 (2)
*p*-value	0.517			0.022		
Gender						
Male	184 (44)	84 (20)	10 (2)	56 (13)	180 (43)	42 (10)
Female	88 (21)	42 (10)	11 (3)	26 (6)	95 (23)	20 (5)
*p*-value	0.173			0.865		
Marital status						
Single	14 (3)	8 (2)	2 (0)	5 (1)	13 (3)	6 (1)
Married	239 (57)	108 (26)	15 (4)	72 (17)	240 (57)	50 (12)
Divorced	9 (2)	9 (2)	3 (1)	5 (1)	12 (3)	4 (1)
Widower	10 (3)	1 (0)	1 (0)	0 (0)	10 (3)	2 (1)
*p*-value	0.090			0.412		
Living situation						
Living alone	101 (24)	52 (12)	5 (1)	29 (7)	110 (26)	19 (5)
Living with others	171 (41)	74 (18)	16 (4)	53 (12)	165 (40)	43 (10)
*p*-value	0.294			0.346		
Education						
High school or less	51 (12)	24 (6)	6 (3)	18 (4)	51 (12)	12 (3)
University education	160 (38)	79 (19)	14 (2)	46 (11)	168 (40)	39 (9)
Higher education	61 (15)	23 (5)	1 (0)	18 (4)	56 (14)	11 (3)
*p*-value	0.319			0.906		
Work status						
Employed	107 (26)	45 (11)	9 (2)	27 (6)	105 (25)	29 (7)
Freelance Job	18 (4)	9 (2)	0 (0)	6 (2)	17 (4)	4 (1)
Retired	120 (29)	56 (13)	8 (2)	39 (9)	122 (29)	23 (5)
Unemployed	27 (6)	16 (4)	4 (1)	10 (2)	31 (8)	6 (2)
*p*-value	0.698			0.806		
Income (SR)						
<2000	19 (5)	14 (3)	4 (1)	10 (2)	22 (5)	5 (1)
2000–5000	22 (5)	8 (2)	4 (1)	7 (1)	20 (5)	7 (2)
5000–7000	14 (3)	8 (2)	1 (1)	3 (1)	13 (3)	7 (2)
7000–10,000	26 (6)	16 (4)	6 (1)	4 (1)	32 (8)	12 (3)
>10,000	191 (46)	80 (19)	6 (1)	58 (14)	188 (45)	31 (7)
*p*-value	0.011			0.038		
BMI category ^2^						
Underweight	2 (0)	2 (0)	0 (0)	0 (0)	2 (0)	2 (0)
Normal weight	53 (13)	26 (6)	5 (1)	23 (5)	54 (13)	7 (2)
Overweight	110 (26)	53 (13)	8 (2)	35 (8)	115 (28)	21 (5)
Obese	107 (26)	45 (11)	8 (2)	24 (6)	104 (25)	32 (8)
*p*-value	0.959			0.068		

^1^ Data presented as number and percentage. ^2^ Self-reported weight and height used to calculate the BMI. The BMI categories are underweight (<18.5 kg/m^2^), normal weight (18.5–24.9 kg/m^2^), overweight (25.0–29.9 kg/m^2^), and obese (≥30 kg/m^2^). Differences between the three groups were assessed via Chi-square test. Abbreviations: SR, Saudi Riyals; BMI, body mass index.

**Table 3:** Dietary intake scoring of different food groups in servings per week (*n* = 419) ^1^.

Variables	Starch	Fruits	Vegetables	Milk and Dairy Products	Red Meat	Poultry	Fish	Legumes
Mean ± SD	8.09 ± 0.24	5.92 ± 0.25	5.57 ± 0.22	5.56 ± 0.23	2.65 ± 0.13	4.34 ± 0.16	1.36 ± 0.08	2.03 ± 0.12
Age (years)								
45–54	7.92 ± 0.35	4.28 ± 0.33	4.77 ± 0.30	5.74 ± 0.36	2.59 ± 0.22	4.79 ± 0.26	1.10 ± 0.12	2.09 ± 0.21
55–64	8.02 ± 0.37	7.25 ± 0.41	6.44 ± 0.37	5.43 ± 0.35	2.57 ± 0.18	3.96 ± 0.24	1.58 ± 0.13	1.89 ± 0.14
≥65	8.90 ± 0.67	7.61 ± 0.70	5.75 ± 0.60	5.32 ± 0.55	3.07 ± 0.43	3.95 ± 0.39	1.61 ± 0.18	2.23 ± 0.30
*p*-value	0.817	<0.001	0.027	0.542	0.267	0.089	<0.001	0.143
Gender								
Male	8.14 ± 0.29	5.52 ± 0.30	5.20 ± 0.26	5.40 ± 0.27	2.94 ± 0.17	4.78 ± 0.21	1.54 ± 0.10	2.25 ± 0.15
Female	7.99 ± 0.40	6.12 ± 0.46	6.30 ± 0.41	5.88 ± 0.43	2.06 ± 0.22	3.48 ± 0.26	1.02 ± 0.12	1.59 ± 0.17
*p*-value	0.903	0.948	0.035	0.649	<0.001	<0.001	<0.001	<0.001
Marital status								
Single	8.06 ± 1.20	3.54 ± 0.81	5.22 ± 0.93	4.14 ± 0.90	1.91 ± 0.45	2.68 ± 0.41	1.35 ± 0.35	1.56 ± 0.31
Married	8.13 ± 0.25	6.08 ± 0.27	5.65 ± 0.24	5.56 ± 0.25	2.76 ± 0.15	4.57 ± 0.18	1.42 ± 0.09	2.10 ± 0.13
Divorced	6.97 ± 0.95	6.12 ± 1.22	4.98 ± 1.08	6.85 ± 0.88	2.59 ± 0.71	3.21 ± 0.72	0.83 ± 0.21	1.50 ± 0.31
Widower	9.00 ± 1.35	5.67 ± 1.60	4.96 ± 1.38	6.29 ± 1.78	0.87 ± 0.17	2.75 ± 0.67	0.41 ± 0.10	1.83 ± 0.60
*p*-value	0.698	0.112	0.655	0.094	0.009	0.002	0.002	0.633
Living situation								
Living alone	8.31 ± 0.38	6.51 ± 0.42	5.78 ± 0.37	5.49 ± 0.37	2.74 ± 0.24	4.25 ± 0.25	1.41 ± 0.12	1.87 ± 0.14
Living with others	7.95 ± 0.31	5.56 ± 0.32	5.45 ± 0.28	5.60 ± 0.29	2.59 ± 0.16	4.40 ± 0.22	1.33 ± 0.11	2.13 ± 0.17
*p*-value	0.423	0.045	0.504	0.888	0.982	0.808	0.194	0.599
Education								
High school or less	7.09 ± 0.50	5.53 ± 0.58	5.74 ± 0.50	5.90 ± 0.58	2.45 ± 0.30	4.63 ± 0.42	1.01 ± 0.10	2.09 ± 0.28
University education	8.13 ± 0.31	5.86 ± 0.33	5.26 ± 0.28	5.55 ± 0.29	2.66 ± 0.18	4.30 ± 0.21	1.38 ± 0.11	2.12 ± 0.17
Higher education	8.91 ± 0.55	6.50 ± 0.56	6.34 ± 0.52	5.26 ± 0.51	2.80 ± 0.31	4.19 ± 0.33	1.64 ± 0.22	1.70 ± 0.16
*p*-value	0.130	0.246	0.167	0.800	0.407	0.919	0.165	0.858
Work status								
Employed	8.59 ± 0.40	5.34 ± 0.41	4.87 ± 0.33	5.66 ± 0.38	2.93 ± 0.25	5.10 ± 0.30	1.20 ± 0.14	2.14 ± 0.21
Freelance Job	8.18 ± 0.96	4.77 ± 0.89	5.50 ± 0.89	5.07 ± 0.84	2.90 ± 0.61	3.66 ± 0.55	1.70 ± 0.37	1.85 ± 0.49
Retired	7.80 ± 0.35	6.80 ± 0.38	6.03 ± 0.35	5.59 ± 0.33	2.59 ± 0.18	3.96 ± 0.21	1.61 ± 0.13	2.04 ± 0.15
Unemployed	7.43 ± 0.65	5.13 ± 0.78	6.23 ± 0.70	5.41 ± 0.79	1.75 ± 0.33	3.66 ± 0.54	0.74 ± 0.08	1.68 ± 0.40
*p*-value	0.547	0.005	0.127	0.830	0.043	0.005	0.001	0.147
Income (SR)								
<2000	7.20 ± 0.77	4.71 ± 0.87	5.79 ± 0.76	5.82 ± 0.94	2.10 ± 0.45	3.56 ± 0.60	0.82 ± 0.13	1.70 ± 0.41
2000–5000	7.01 ± 0.85	3.93 ± 0.86	5.54 ± 0.77	5.55 ± 0.87	2.14 ± 0.49	4.02 ± 0.60	1.52 ± 0.42	1.63 ± 0.33
5000–7000	10.2 ± 0.97	5.65 ± 1.24	4.30 ± 0.82	7.02 ± 1.02	1.80 ± 0.27	3.87 ± 0.66	1.10 ± 0.23	2.76 ± 0.62
7000–10,000	7.42 ± 0.68	6.31 ± 0.79	5.13 ± 0.71	5.81 ± 0.71	2.11 ± 0.39	3.66 ± 0.43	0.89 ± 0.14	1.44 ± 0.20
>10,000	8.28 ± 0.29	6.28 ± 0.30	5.73 ± 0.27	5.36 ± 0.27	2.94 ± 0.17	4.65 ± 0.20	1.52 ± 0.11	2.16 ± 0.15
*p*-value	0.115	0.004	0.438	0.594	0.001	0.033	0.012	0.017
BMI category ^2^								
Underweight	4.75 ± 0.75	5.88 ± 3.04	5.25 ± 2.98	5.13 ± 3.04	1.37 ± 0.37	4.75 ± 3.09	0.62 ± 0.23	1.50 ± 0.28
Normal weight	7.64 ± 0.53	5.76 ± 0.58	5.22 ± 0.48	5.30 ± 0.51	2.55 ± 0.29	3.71 ± 0.30	1.35 ± 0.12	1.72 ± 0.22
Overweight	7.69 ± 0.37	5.91 ± 0.38	5.88 ± 0.35	5.29 ± 0.36	2.67 ± 0.23	4.28 ± 0.25	1.57 ± 0.17	2.15 ± 0.20
Obese	8.83 ± 0.39	6.02 ± 0.43	5.44 ± 0.36	6.00 ± 0.38	2.70 ± 0.22	4.74 ± 0.29	1.17 ± 0.09	2.07 ± 0.19
*p*-value	0.094	0.929	0.657	0.356	0.873	0.371	0.487	0.636

^1^ Dietary intake scores were calculated based on the frequency of intake of each food group and the portion size consumed each time and were rated on a 7-point scale. A score of 0 was given when no consumption was reported, a score of 0.5 when <1 serving/week was reported, a score of 1 when 1 serving/week was reported, 2 for 2–3 servings/week, 4 for 4–6 servings/week, 7 for 7 servings/weeks or 1 serving/day, and a score of 14 when ≥14 servings/week or more or ≥2 servings/day. ^2^ Self-reported weight and height used to calculate the BMI. The BMI categories are underweight (<18.5 kg/m^2^), normal weight (18.5–24.9 kg/m^2^), overweight (25.0–29.9 kg/m^2^), and obese (≥30 kg/m^2^). Differences between the groups were assessed via Kruskal–Wallis test. Abbreviations: SD, standard deviation; SR, Saudi Riyals; BMI body mass index.

## Data Availability

The data used to support the findings of this study are available from the corresponding author upon request.
